# Impact of severe dysphagia on overall survival after percutaneous endoscopic gastrostomy

**DOI:** 10.1038/s41598-025-88097-y

**Published:** 2025-01-29

**Authors:** Kazumi Shimamoto, Ryota Matsui, Yorihiro Nishiyama, Kyohei Nishino, Hiromitsu Ban

**Affiliations:** 1 Department of Gastroenterology, Omi Medical Center, 1660 Yabase-cho, Kusatsu, 525- 8585 Shiga Japan; 2 Department of Gastroenterological Surgery, The Cancer Institute Hospital of JFCR, 3-8-31 Ariake, Koto-ku, Tokyo, 135-8550 Japan; 3 Department of Upper Gastrointestinal Surgery, Juntendo University Hospital, 3-1-3 Hongo, Bunkyo- ku, Tokyo, 113-8431 Japan

**Keywords:** Gastroenterology, Medical research, Risk factors

## Abstract

In this study, we investigated the effect of severe dysphagia on the overall survival of patients who underwent PEG. A cohort of patients who underwent PEG between April 2016 and April 2021 was retrospectively analyzed. The Hyodo–Komagane score was used to evaluate swallowing via endoscopy. Patients with a Hyodo–Komagane score ≤ 8 were defined as having moderate dysphagia, whereas those with a score ≥ 9 were defined as having severe dysphagia. The primary outcome was overall survival. The prognostic factors were identified using multivariate analysis with the Cox proportional hazards model. Values of *p* < 0.05 were considered statistically significant. Among the 107 patients, 60 (56.1%) were classified into the moderate dysphagia group and 47 (43.9%) into the severe dysphagia group. The median follow-up period was 16.7 months. The overall survival was significantly worse in the severe group than in the moderate group (*p* < 0.0001). A multivariate analysis revealed that severe dysphagia was an independent poor prognostic factor (hazard ratio, 2.956; 95% confidence interval, 1.592–5.489; *p* < 0.001). Aspiration-related pneumonia was most common causes of death after PEG. Severe dysphagia was identified as an independent poor prognostic factor for survival in patients who underwent PEG.

**Supplementary Information:**

The online version contains supplementary material available at 10.1038/s41598-025-88097-y.

## Introduction

Percutaneous endoscopic gastrostomy (PEG), introduced by Gauderer et al. in 1980, is widely used as an enteral feeding access for artificial nutrition^1^. PEG for mid- to long-term nutritional management is superior to nasogastric tube feeding in terms of patient tolerability and social acceptance^2–4^. Furthermore, PEG reportedly causes gastroesophageal reflux disease and aspiration pneumonia less frequently than nasogastric tube feeding, and the effectiveness of nutritional intervention with PEG is superior to that of nasogastric tube^4,5^. Therefore, when nutritional intake is inadequate and artificial enteral nutrition is required for more than 2 or 3 weeks, nutritional support should be provided through gastrostomy^6^.

The impact of dysphagia on their long-term prognosis after PEG is unclear. Subclinical aspiration can occur even in patients who do not orally consume food, and high dysphagia severity is correlated with an increased risk of developing aspiration pneumonia. In Japan, evaluation of swallowing function is recommended before PEG is performed. Therefore, this study may help determine patient prognosis after PEG based on the severity of dysphagia. If the prognosis is poor, severe dysphagia may not be an ideal indication for PEG.

In this study, we aimed to investigate the association between the severity of dysphagia and post-PEG survival. We hypothesized that severe dysphagia is a poor prognostic factor for post-PEG survival. If it is established that severe dysphagia is associated with significantly shorter survival after PEG, this information will be crucial in determining whether PEG should be performed.

## Materials and methods

### Study participants

Patients who underwent PEG for enteral nutritional access at Omi Medical Center between April 2016 and April 2021 were eligible for the study. All patients who underwent endoscopy for the evaluation of swallowing function before and after PEG were included, and those who did not undergo endoscopy or whose swallowing function could not be evaluated were excluded. This study included only patients who did not orally consume food after PEG.

All experimental protocols described in this study were approved by the Institutional Ethical Review Committee of Omi Medical Center (*authorization number*:2023-0046). The study adhered to the ethical guidelines of the Japan Ministry of Health, Labour and Welfare for Medical and Health Research Involving Human Subjects and conformed to the principles outlined in the Declaration of Helsinki. The opt-out recruitment method was used, which allowed the patients to decline participation. Informed consent was obtained from all participants.

### Definition of dysphagia

The Hyodo–Komagane scoring method was used to endoscopically evaluate swallowing function (Table 1)^7,8^. A higher Hyodo–Komagane score indicated a more severe decline in swallowing function. Since oral intake was considered challenging at a score ≥ 9^7,8^, patients with a Hyodo–Komagane score ≤ 8 were categorized into the moderate dysphagia group, whereas those with a score ≥ 9 were classified into the severe dysphagia group.

**Table 1:** Hyodo–komagane scoring rubric.

A: Salivary pooling in vallecula and piriform sinuses
0 No pooling
1 Pooling only at the vallecula
2 Pooling in vallecula and piriform sinuses and no penetration1 into larynx
3 Pooling in vallecula and piriform sinuses and penetration into larynx
B: The response of glottal closure reflex induced by touching the epiglottis with the endoscope
0 Marked reflex by one touching
1 Slow and/or weak reflex by one touching
2 Reflex by two or three touchings
3 No reflex despite three touchings
C: The location of the bolus at the time of swallow onset assessed by “white-out"2 following swallowing of test jelly
0 Pharyngeal
1 Vallecula
2 Piriform sinuses
3 No swallowing
D: The extent of pharyngeal clearance after swallowing of test jelly
0 No residues
1 Pharyngeal residues remain, but are absent after swallowing is attempted two or three times
2 Pharyngeal residues remain, but do not penetrate into larynx
3 Pharyngeal residues remain and penetrate into larynx

1　When saliva or test jelly enters the glottis (opening to the trachea) and moves as far as the vestibule above the true vocal folds, this is termed as “penetration”.

2　“White-out” is defined as the period when the videoendoscopic image is obscured owing to pharyngeal closure.

Total score = (A + B + C + D) = 0–12.

### Outcome

The primary outcome was overall survival (OS) after PEG, which was defined as the time from the date of PEG administration to the date of death. Patients who were not followed up at the hospital were investigated by hospital staff to confirm whether the patients were alive or dead. Patients for whom survival status was unclear were also excluded.

### Statistical analyses

Fisher’s exact test was used to compare patient demographics between the two groups, and the log-rank test was used to compare OS and generate Kaplan–Meier survival curves. We performed a multivariate analysis to identify independent poor prognostic factors and incorporated variables with values of *p* < 0.15 from the univariate analysis obtained using the Cox proportional hazards regression model. All statistical analyses were performed using EZR software (Saitama Medical Center, Jichi Medical University, Japan). Values of *p* < 0.05 were considered statistically significant.

### Covariates

The variables used for prognostic comparison included the severity of dysphagia, sex, age, body mass index, serum albumin, total cholesterol (TC), total lymphocyte count (TLC), C-reactive protein (CRP), Controlling Nutritional Status (CONUT) score, performance status, Mini Nutritional Assessment Short-Form score, and comorbidities. For each cut-off value, we used values that were previously reported as being related to prognosis^9–12^.

## Results

### Patient demographics

Patient demographics are summarized in Table 1. Among the 107 patients, 60 (56.1%) were categorized into the moderate dysphagia group and 47 (43.9%) into the severe dysphagia group. The median Hyodo-Komagane score was 7.62 (IQR 5.5–10). The proportion of male patients was significantly higher (*p* < 0.001) and the median TC level was significantly lower (*p* = 0.005) in the severe dysphagia group than in the moderate dysphagia group. In the background, the proportion of traumatic brain injury was statistically significantly higher (*p* < 0.05) in the severe dysphagia group.(Table 2).

**Table 2:** Characteristics of study patients.

	Moderate dysphagia (*N* = 60)	Severe dysphagia (*N* = 47)	*P* value
Age (years), mean ± SD	80.82 ± 10.97	80.79 ± 7.75	0.553
Sex Male Female	19 (31.7%) 41 (68.3%)	35 (74.5%) 12 (25.5%)	< 0.001
BMI (kg/m^2^), mean ± SD	17.57 ± 3.1	17.74 ± 3.65	0.803
Serum albumin (g/dL) median [IQR]	2.80 [2.50, 3.00]	2.80 [2.55, 3.10]	0.573
TC (mg/dL), median [IQR]	165.0 [143.5, 199.0]	149.0 [115.3, 168.0]	0.005
TLC (cells/mm^2^), median [IQR]	1470.2 [908.9, 1995.8]	1402.2 [945.3, 1939.2]	0.742
CRP (mg/dL), median [IQR]	0.45 [0.19, 2.10]	0.98 [0.29, 1.74]	0.427
CONUT, median [IQR]	5.63 [4.00, 7.50]	5.93 [4.00, 8.00]	0.569
PS, median [IQR]	3.00 [3.00, 4.00]	3.00 [3.00, 4.00]	0.665
MNA-SF, median [IQR]	8.00 [6.00, 9.00]	7.00 [5.00, 9.00]	0.631
Comorbidity Diabetes	7 (11.7%)	9 (19.1%)	0.413
COPD	2 (3.3%)	1 (2.1%)	1.000
CHF	11 (18.3%)	10 (21.3%)	0.808
CKD	1 (1.7%)	2 (4.3%)	1.000
Hypertension	20 (33.3%)	29 (61.7%)	0.006
Background (cause of dysphagia) 1.Diseases CVD	27(45.0%)	21 (44.7%)	1.000
ND	9(15.0%)	6 (12.8%)	0.787
TBI	0 (0%)	4 (8.5%)	0.035
Tumor (Neck cancer, Neurogenic tumor, Lung cancer)	2 (3.3%)	6 (12.8%)	0.134
2.Others (Age-related change, Dementia)	22 (36.7%)	10 (21.3%)	0.094

*SD* standard deviation, *BMI* body mass index, *IQR* interquartile range, *TC* total cholesterol, *TLC* total lymphocyte count, *CRP* C-reactive protein, *CONUT* Controlling Nutritional Status, *PS* performance status, *MNA-SF* Mini Nutritional Assessment Short-Form, *COPD* chronic obstructive pulmonary disease, *CHF* chronic heart failure, *CKD c*hronic kidney disease, *CVD* cerebrovascular diseases, *ND* neurodegenerative diseases, *TBI* Traumatic brain injury.

### Comparison of overall survival

**Fig. 1:**
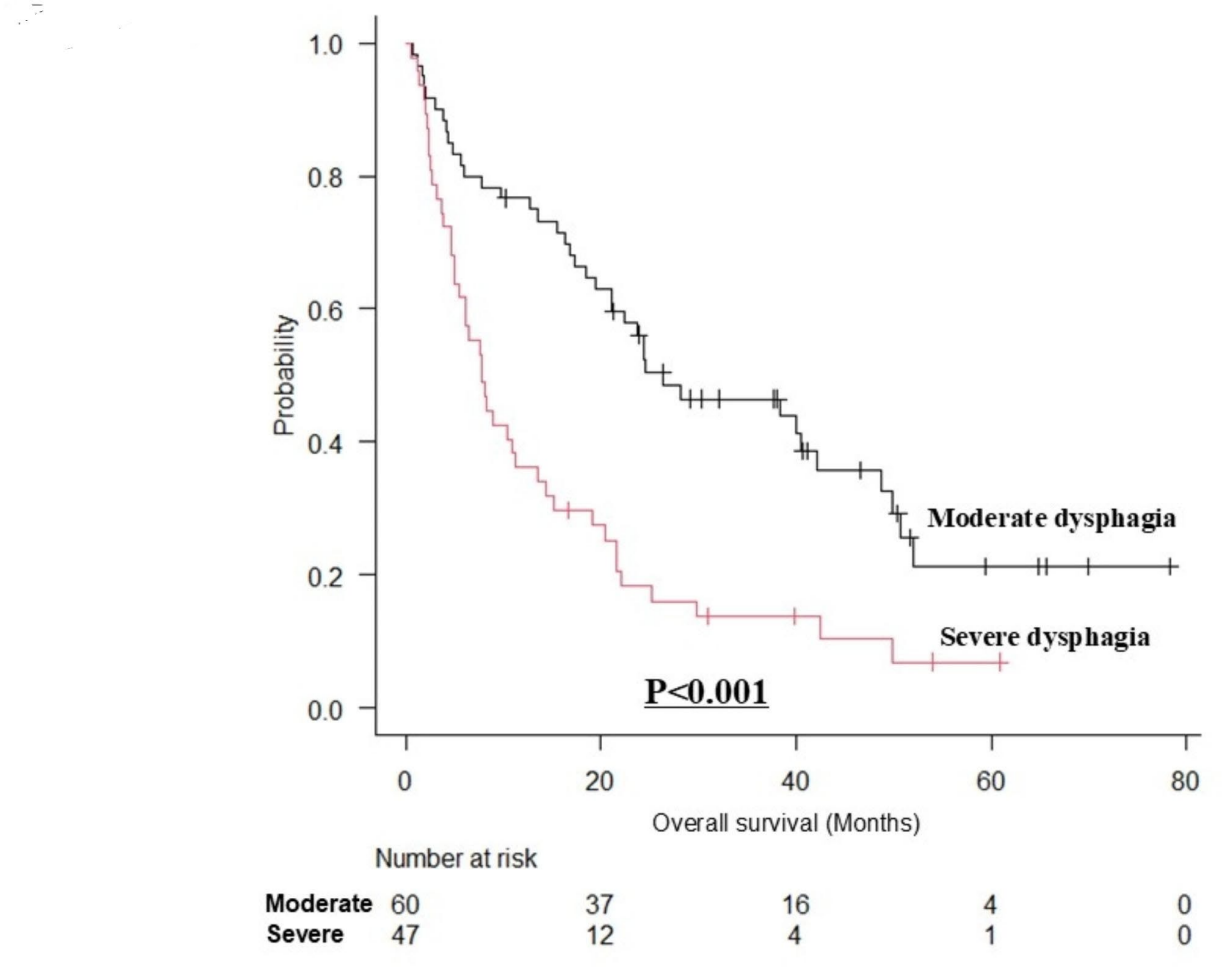
Survival curves for Hyodo–Komagane scores ≤ 8 (moderate dysphagia group) versus ≥ 9 (severe dysphagia group).

**Fig. 2:**
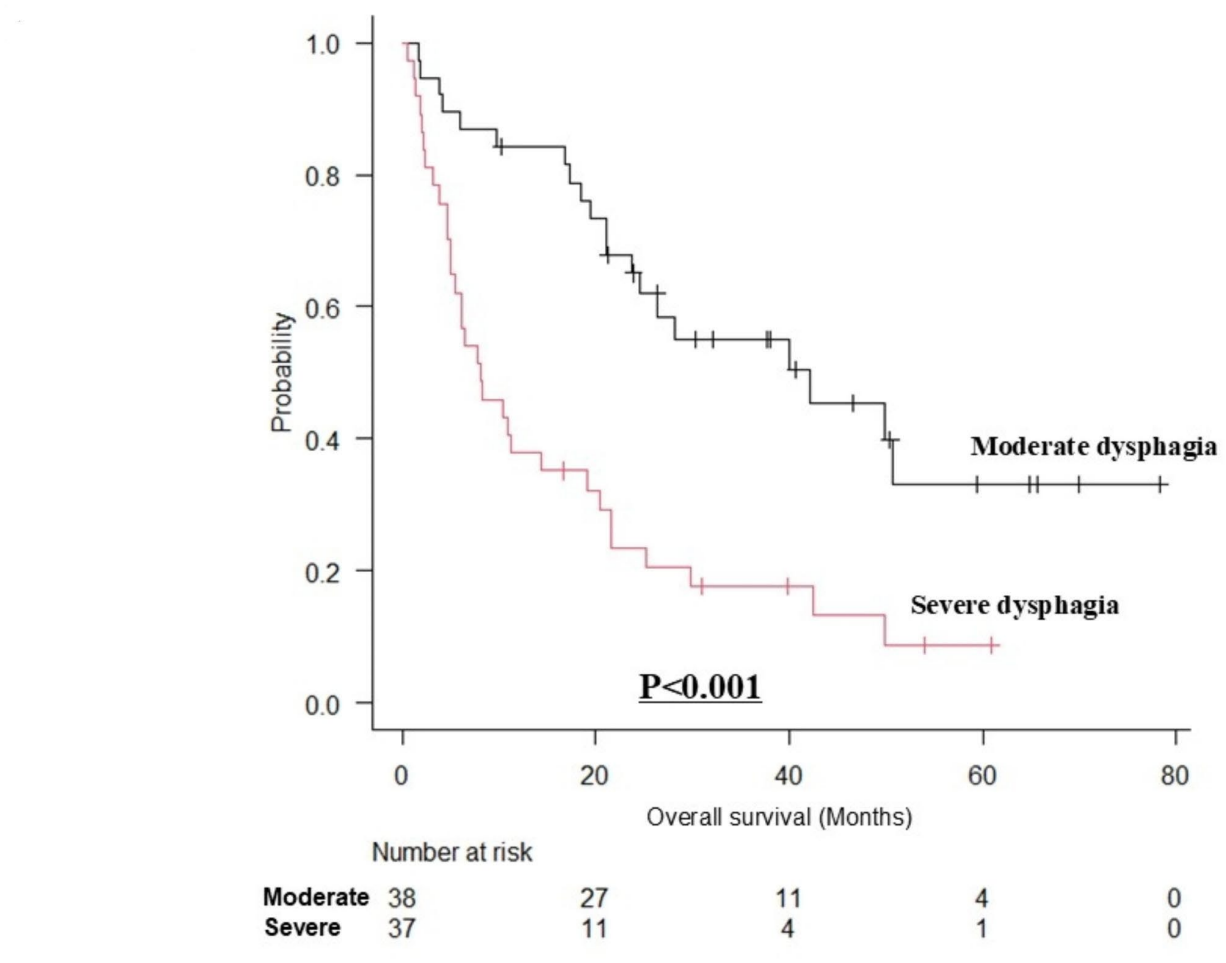
Survival curves for moderate dysphagia group versus severe dysphagia group (with obvious desease).

**Fig. 3:**
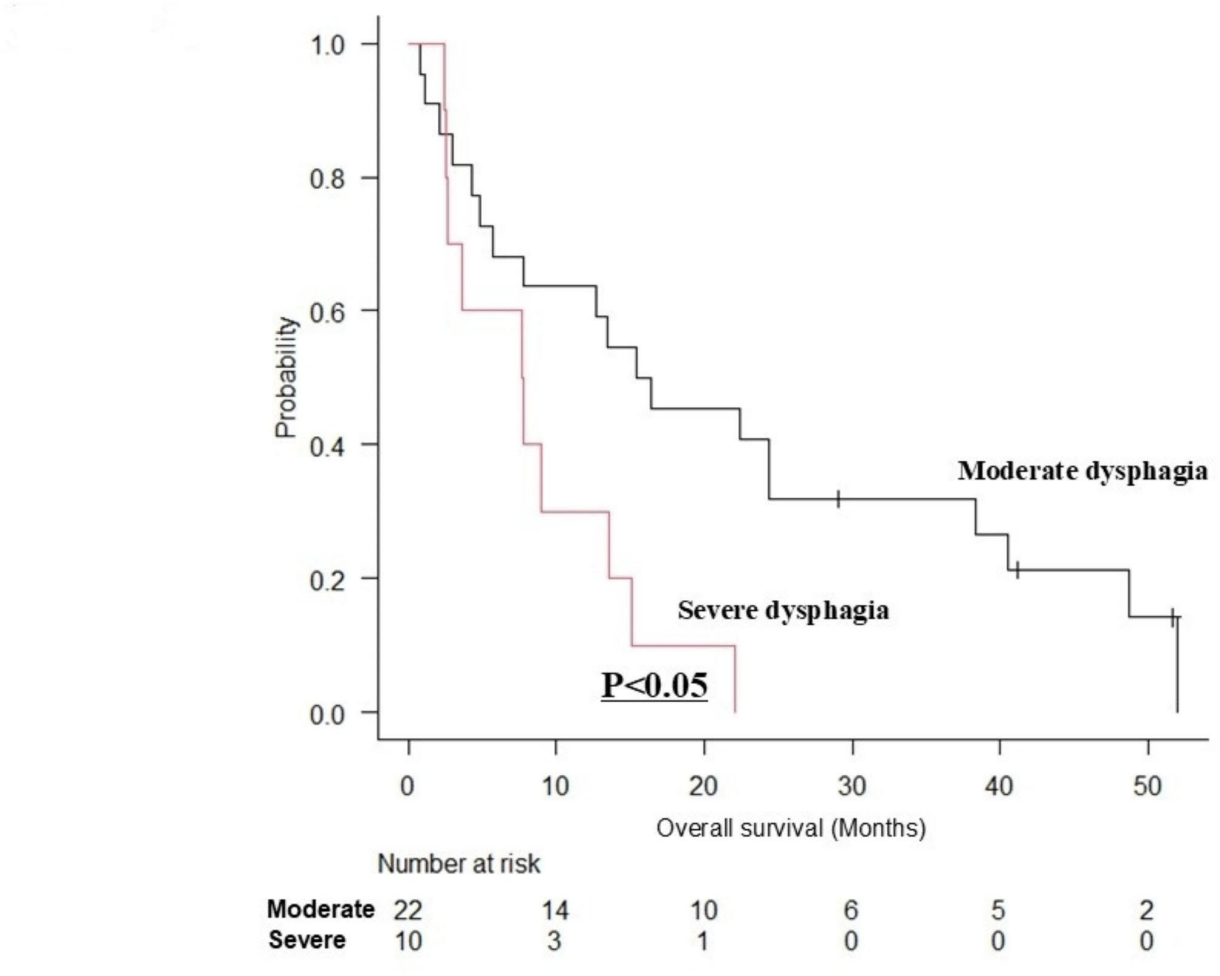
Survival curves for moderate dysphagia group versus severe dysphagia group (with physical function decline due to aging or dementia).

The median follow-up period was 16.7 (interquartile range [IQR], 5.2–30.2) months. The OS was significantly worse in the severe dysphagia group than in the moderate dysphagia group (*p* < 0.0001) (Fig. 1). The median survival time was 23.8 (IQR, 11.9–40.0) months for the moderate dysphagia group and 7.7 (IQR, 3.7–19.5) months for the severe dysphagia group. The 1-year survival rates in the moderate and severe dysphagia groups were 76.7% and 36.2%, respectively. In the group with obvious disease (moderate dysphagia *n* = 38, severe dysphagia *n* = 37), the prognosis was statistically significantly worse in the severe dysphagia group than in the moderate dysphagia group (*p* < 0.001) (Fig. 2). The median survival time was 26.4 (IQR, 18.7–41.7) months for the moderate dysphagia group and 8.1 (IQR, 4.6–21.5) months for the severe dysphagia group. In the group with physical function decline due to aging or dementia (moderate dysphagia *n* = 22, severe dysphagia *n* = 10), the prognosis was statistically significantly worse in the severe dysphagia group than in the moderate group (*p* < 0.05) (Fig. 3). The median survival time was 15.9 (IQR, 5.1–36.1) months for the moderate group and 7.7 (IQR, 2.9–12.4) months for the severe group.

### Prognostic factors for overall survival

The results of the analysis of the prognostic factors related to OS after PEG are presented in Table 3. The univariate analysis revealed that severe dysphagia (*p* < 0.001), male sex (*p* = 0.002), TC < 160 mg/dL (p *=* 0.007), TLC < 1200 cells/mm^2^ (p *=* 0.011), and CONUT score ≥ 5 (p *=* 0.002) were significant poor prognostic factors associated with OS after PEG. The multivariate analysis revealed that severe dysphagia was an independent poor prognostic factor (hazard ratio, 2.956; 95% confidence interval, 1.592–5.489, *p* < 0.001).

**Table 3:** Analyses of prognostic factors for overall survival after PEG.

	Univariate analysis HR 95%CI *P* value			Multivariate analysis HR 95%CI *P* value		
Dysphagia Moderate Severe	1 2.459	1.572–3.846	<0.001	1 2.956	1.592–5.489	<0.001
Age (years) < 85 ≥ 85	1 1.295	0.827–2.027	0.259			
Sex Female Male	1 2.052	1.308–3.221	0.002	1 0.758	0.386–1.488	0.421
BMI (kg/ m^2^) ≥ 18.5 < 18.5	1 1.327	0.837–2.103	0.229			
Serum albumin (g/dL) ≥ 3.0 < 3.0	1 1.493	0.915–2.437	0.109	1 0.766	0.382–1.536	0.452
TC (mg/dL) ≥ 160 < 160	1 1.862	1.183–2.931	0.007	1 1.382	0.764–2.499	0.285
TLC (cells/mm^2^) ≥ 1200 < 1200	1 1.780	1.140–2.780	0.011	1 1.718	0.997–2.963	0.051
CRP (mg/dL) < 0.5 ≥ 0.5	1 1.52	0.970–2.360	0.067	1 1.095	0.964–1.243	0.162
CONUT score < 5 ≥ 5	1 2.287	1.355–3.861	0.002	1 1.974	0.861–4.525	0.108
DM Absent Present	1 1.61	0.860–3.025	0.135	1 1.173	0.584–2.359	0.654

*HR* hazard ratio, *CI c*onfidence interval, *BMI* body mass index, *TC* total cholesterol, *TLC* total lymphocyte count, *CRP* C-reactive protein, *CONUT* Controlling Nutritional Status, *DM* diabetes mellitus.

### Comparison of cause of death

Table 4 compares the causes of death between the groups. During the observation period, 39 of the 60 patients (65.0%) in the moderate dysphagia group and 42 of the 47 patients (89.4%) in the severe dysphagia group died. Among the 81 patients who died after PEG, aspiration-related pneumonia was the most common cause (57 [70.4%]). Aspiration-related pneumonia was considerly more common in the severe dysphagia group than in the moderate dysphagia group (61.7% vs. 46.7%, respectively; *p* = 0.172), though the defference was not statistically significant.

**Table 4:** Causes of death in the moderate or severe dysphagia group.

Cause of death	Moderate dysphagia (*N* = 60)	Severe dysphagia (*N* = 47)	*P* value
Aspiration-related pneumonia	28 (46.7%)	29 (61.7%)	0.172
Malignant tumor	2 (3.3%)	3 (6.4%)	0.652
Heart failure	3 (5.0%)	2 (4.3%)	1.000
Unknown	1 (1.7%)	4 (8.5%)	0.166
Cerebral infarction	1 (1.7%)	0 (0.0%)	1.000

## Discussion

In this study, we investigated the relationship between the severity of dysphagia and OS after PEG. Severe dysphagia was identified as a poor prognostic factor for OS in patients after PEG. Several new findings emerged from this study. First, severe dysphagia was identified as an independent poor prognostic factor for OS in patients after PEG. Second, the comparison of the moderate and severe dysphagia groups revealed no differences in background disease or nutritional status except for traumatic brain injury and sex. Third, aspiration-related pneumonia, the most common cause of death, was more common in the severe dysphagia group than in the moderate dysphagia group. To our knowledge, this is the first study to demonstrate that severe dysphagia is a poor prognostic factor for OS in patients after PEG.

The multivariate analysis based on the Cox proportional hazards model revealed that severe dysphagia, defined as a Hyodo–Komagane score ≥ 9, was an independent poor prognostic factor for OS in patients after PEG. In the present study, prognosis after PEG was poor with a shorter survival in the severe dysphagia group than in the moderate dysphagia group. Regarding the association between dysphagia and prognosis, Patel et al. reported that, among 88 million adult hospitalized patients aged ≥ 45 years, 2.7 million with dysphagia had a 1.7 times higher hospitalization mortality rate^13^, and Light et al. suggested that a history of aspiration pneumonia is a poor prognostic factor in post-PEG patients^10^. These studies suggest that the degree of dysphagia may influence prognosis, which is consistent with our findings.

The comparison of the moderate and severe dysphagia groups demonstrated no differences in background disease, nutritional status, or other factors except for traumatic brain injury and sex. This indicates that no significant differences exist in the prognostically relevant indices for PEG except for the severity of dysphagia. Several studies have suggested that serum albumin^14–17^, TLC^17,18^, TC^19^, and CRP levels are prognostic factors for OS after PEG^17^. Lang et al. investigated 30- or 60-day survival rates after PEG in 502 patients and found that a serum albumin level < 3.0 g/dL is a significant risk factor for early death^11^. In this study, despite evaluating prognosis using cut-off values from previous studies, the multivariate analysis revealed that a Hyodo–Komagane score ≥ 9 was the poorest prognostic factor for OS among other factors in patients who underwent PEG.

Aspiration-related pneumonia, the most common cause of death after PEG (70.4%), was more common in the severe dysphagia group than in the moderate group. Although there was no statistically significant difference in the frequency of aspiration pneumonia when comparing the causes of death in the groups with moderate and severe dysphagia, it is clear that aspiration is a common cause of death in both groups, and in the group with severe dysphagia, there is a possibility of death due to aspiration occurring at an earlier stage after PEG. The severity of dysphagia reflects the severity of the background disease and the severity of the general condition, which affects survival after PEG. A meta-analysis by Kondwani et al. demonstrated that 42% of patients developed dysphagia following stroke, with a 4.08-fold increased risk of pneumonia and a 4.07-fold increased mortality rate^20^. The present study demonstrated that the majority of patients had post-stroke dysphagia.

In this study, Hyodo–Komagane score was an objective measure of dysphagia, exhibiting a clear correlation with a poor prognosis. Swallowing function should be evaluated before PEG is performed, and endoscopy is highly useful because it is easy to perform. During endoscopic swallowing function evaluations, an objective rather than subjective judgment should be made. Based on the results of this study, it is imperative that clinicians communicate to patients or their families that a score ≥ 9 indicates a poor prognosis despite PEG.

This study had several limitations. First, it was a single-center retrospective study; therefore, further multicenter prospective studies are required to validate the generalizability of its results. Second, its sample size was relatively small. However, despite the small sample size, we demonstrated that severe dysphagia is an important prognostic factor among patients who undergo PEG. This is the first study to demonstrate that severe dysphagia is an independent poor prognostic factor for OS in patients after PEG, which is of high clinical value. In patients with severe dysphagia after PEG, improving swallowing function is often challenging and associated with a poor prognosis. These results suggest that patients with severe dysphagia should be informed that long-term survival may be difficult before deciding whether to undergo PEG. Aspiration prevention surgery should be considered as a treatment option for patients with severe dysphagia who strongly desire to continue oral intake even if they are unable to speak or who want to avoid death due to aspiration as much as possible. Even if the patient has severe dysphagia, if they are in a lot of pain from a nasogastric tube, it is not wrong to perform PEG to improve their quality of life. If a patient with severe dysphagia does not complain of any pain due to nasogastric tube, it may be acceptable to continue feeding via a nasogastric tube without PEG, which may cause complications and postoperative pain, or to choose peripheral infusion.

## Conclusion

In this study, patients with severe dysphagia experienced significantly shorter survival after PEG than that of patients with moderate dysphagia. The multivariate analysis revealed that severe dysphagia was an independent poor prognostic factor for OS in patients after PEG.

## Electronic supplementary material

Below is the link to the electronic supplementary material.

Supplementary Material 1.

Supplementary Material 2.

Supplementary Material 3.

Supplementary Material 4.

Supplementary Material 5

## Data Availability

Sequence data that support the findings of this study have been deposited in figshare.10.6084/m9.figshare.25679520.
