# Supplementary figures and images for: Impact of severe dysphagia on overall survival after percutaneous endoscopic gastrostomy
Source: Sci Rep. 2025 Jan 29;15:3617. doi: 10.1038/s41598-025-88097-y (PMC11775191; doi:10.1038/s41598-025-88097-y)

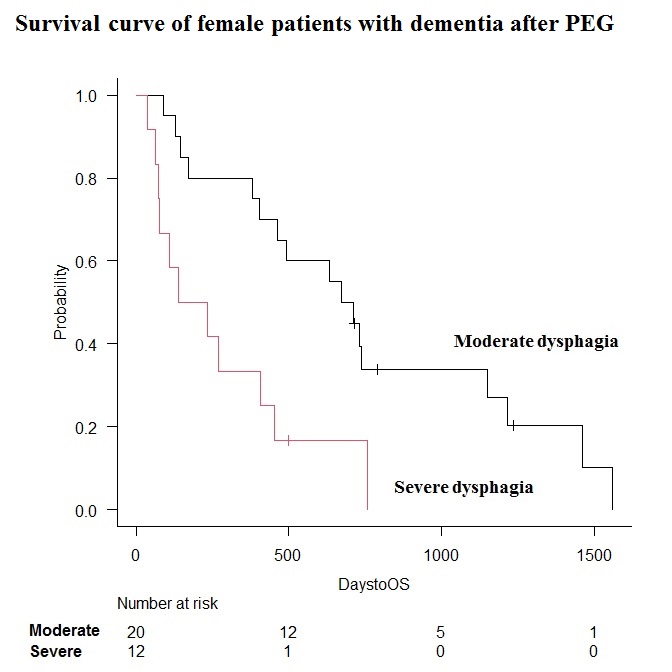

Supplement: Supplementary file 1 — Supplementary Material 1. [file 41598_2025_88097_MOESM1_ESM.jpg]

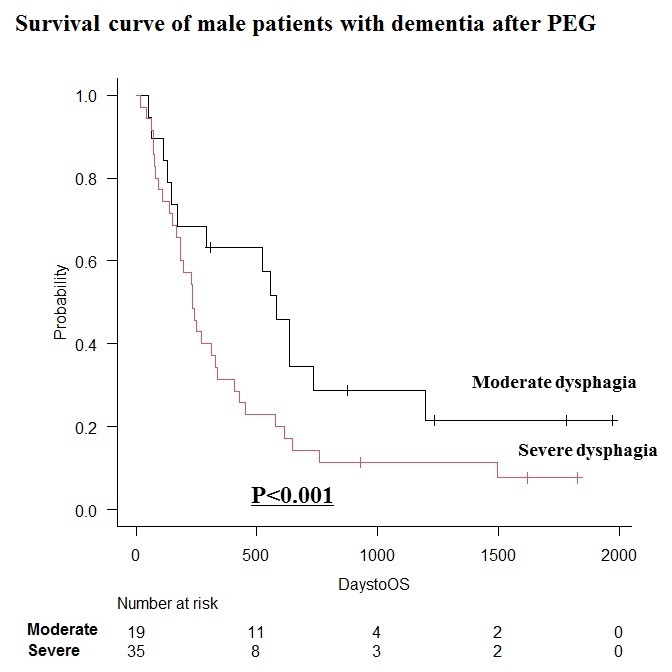

Supplement: Supplementary file 2 — Supplementary Material 2. [file 41598_2025_88097_MOESM2_ESM.jpg]

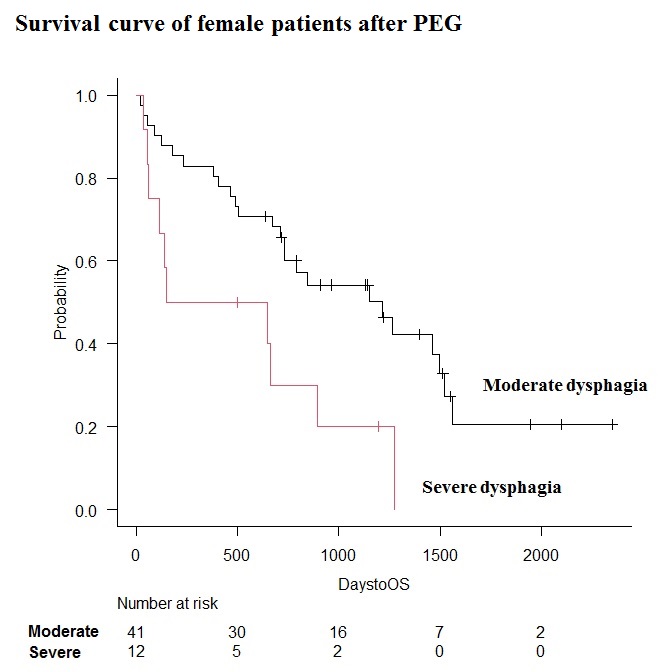

Supplement: Supplementary file 3 — Supplementary Material 3. [file 41598_2025_88097_MOESM3_ESM.jpg]

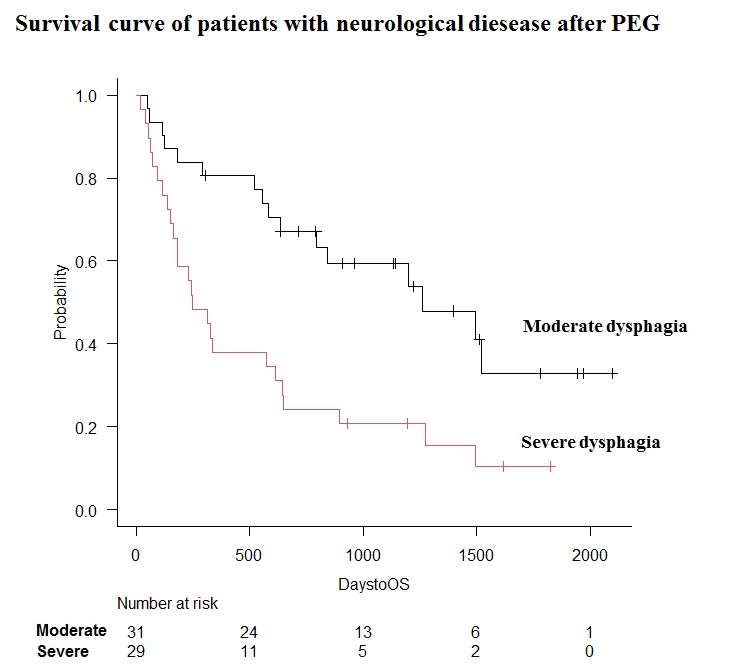

Supplement: Supplementary file 4 — Supplementary Material 4. [file 41598_2025_88097_MOESM4_ESM.jpg]

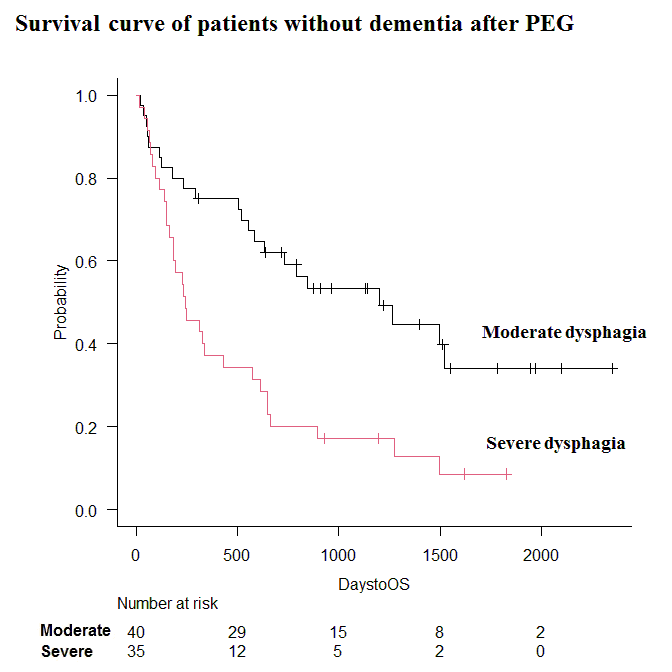

Supplement: Supplementary file 5 — Supplementary Material 5 [file 41598_2025_88097_MOESM5_ESM.bmp]
